# Conservative surgery in multimodal therapy for pelvic rhabdomyosarcoma in children.

**DOI:** 10.1038/bjc.1994.438

**Published:** 1994-11

**Authors:** A. Atra, H. C. Ward, K. Aitken, M. Boyle, C. Dicks-Mireaux, P. G. Duffy, C. D. Mitchell, P. N. Plowman, P. G. Ransley, J. Pritchard

**Affiliations:** Department of Haematology and Oncology, Hospitals for Sick Children, London, UK.

## Abstract

Twenty-six previously untreated children, median age 3.4 years, with pelvic rhabdomyosarcoma (RMS) were seen between 1983 and 1988. Fourteen were girls. The planned strategy was to conserve pelvic organs, especially the bladder, by using primary chemotherapy, conservative surgery and, in most cases, radiotherapy. With a median follow-up of 71 months (range 34-103 months) overall survival was 73%, with no treatment-related death. The bladder salvage rate of 88% in survivors with bladder base/prostate primaries was much higher than that reported by the United States Intergroup Rhabdomyosarcoma Studies (IRS), though many of the preserved bladders did not function normally. We identified problems with both radiological and histological off-treatment monitoring. The overall accuracy of computerised tomographic (CT) scanning for prediction of tumour recurrence was only 81%, and endoscopic biopsies proved misleading in four of the ten bladder base/prostate patients monitored by serial cystoscopy. We conclude that a higher cure rate can be achieved by using intensive chemotherapy/radiotherapy and conservative surgery to treat children with pelvic RMS. Factors that might contribute to our favourable bladder salvage results, compared with those of the IRS, include (a) the fact that one of two specialist surgeons monitored and operated on all these patients and (b) our increasing awareness, during the study, that post-chemotherapy/radiotherapy histopathology and pelvic CT scan appearances may be misleading. Referral to paediatric centres with special experience of pelvic RMS may help raise the rate of bladder salvage in these children.


Br. J. Cancer (1994), 70, 1004–1008 © Macmillan Press Ltd., 1994

### Conservative surgery in multimodal therapy for pelvic rhabdomyosarcoma in children

A. Atra, H.C. Ward*, K. Aitken, M. Boyle†, C. Dicks-Mireaux, P.G. Duffy, C.D. Mitchell, P.N. Plowman, P.G. Ransley & J. Pritchard

*Departments of Haematology and Oncology, Histopathology, Radiology and Urology, The Hospitals for Sick Children, Great Ormond Street, London WC1N 3JH, UK.*

**Summary** Twenty-six previously untreated children, median age 3.4 years, with pelvic rhabdomyosarcoma (RMS) were seen between 1983 and 1988. Fourteen were girls. The planned strategy was to conserve pelvic organs, especially the bladder, by using primary chemotherapy, conservative surgery and, in most cases, radiotherapy. With a median follow-up of 71 months (range 34–103 months) overall survival was 73%, with no treatment-related death. The bladder salvage rate of 88% in survivors with bladder base/prostate primaries was much higher than that reported by the United States Intergoup Rhabdomyosarcoma Studies (IRS), though many of the preserved bladders did not function normally. We identified problems with both radiological and histological off-treatment monitoring. The overall accuracy of computerised tomographic (CT) scanning for prediction of tumour recurrence was only 81%, and endoscopic biopsies proved misleading in four of the ten bladder base/prostate patients monitored by serial cystoscopy. We conclude that a higher cure rate can be achieved by using intensive chemotherapy/radiotherapy and conservative surgery to treat children with pelvic RMS. Factors that might contribute to our favourable bladder salvage results, compared with those of the IRS, include (a) the fact that one of two specialist surgeons monitored and operated on all these patients and (b) our increasing awareness, during the study, that post-chemotherapy/radiotherapy histopathology and pelvic CT scan appearances may be misleading. Referral to paediatric centres with special experience of pelvic RMS may help raise the rate of bladder salvage in these children.

The cure rate for pelvic rhabdomyosarcoma in children is now approximately 70%, although survival depends on the tumour stage at diagnosis and the exact site of origin within the pelvis (Flamant *et al.*, 1990; Raney *et al.*, 1990). Primary tumours are not always controlled by established treatment regimens, and some children die from local regrowth of tumour with or without disseminated disease. Despite this difficulty with 'local control', most treatment teams now try to limit the morbidity of surgery. Total cystectomy/cystoprostatectomy, which results in loss of bladder and sexual function, may be avoided in some children by performing surgery after several months of chemotherapy. The 'shrunken' tumour may then be resectable without ablating pelvic organs and radiotherapy used to treat any residual disease. Only 11 of 20 children with pelvic rhabdomyosarcoma treated at this hospital with chemotherapy, radiotherapy and surgery between 1976 and 1983 survived, and only six retained their bladders (Broecker *et al.*, 1988). After 1983, we formally adopted a conservative surgical policy to try to spare children total cystoprostatectomy or total cystectomy.

The main aim of this study was to assess the results of multimodal therapy, including this conservative surgical policy, in a paediatric hospital with close liaison between paediatric oncologists, urologists, histopathologists and radiologists. Supplementary aims were to determine the value of serial computerised tomographic (CT) scanning and endoscopic biopsies in the assessment of local tumour control

### Patients, Investigations and Monitoring

We studied all 26 children with pelvic rhabdomyosarcoma, excluding those with paratesticular tumours, referred to this hospital between 1983 and 1988 (Table I). In five cases surgical resection, not involving ablation of pelvic viscera, had been attempted elsewhere but none of the children had received chemotherapy or radiotherapy. Age at presentation ranged from 3 months to 14.9 years (median 3.4 years) and 14 patients were girls.

The exact sites of the primary tumours were: bladder base/prostate (13 patients), bladder dome (3), uterus/vagina (5) and pelvic wall (5). Initial investigations included posteroanterior and lateral chest radiographs, abdominal ultrasound, CT scanning of chest and pelvis, radioisotope bone scan and bilateral iliac bone marrow aspiration. These studies indicated that 22 patients had group 3 disease (unresectable local tumour), two group 4 and one each group 1 and 2. In the case of 'endophytic' tumours of the bladder/prostate and vagina accessible by endoscopy, examination under anaesthetic and biopsies were carried out by one of the two surgeons (P.G.R. and P.G.D.) involved in the study. Histological subtypes were embryonal (24) and alveolar (2).

Reassessments involved sequential contrast-enhanced CT scans of the pelvis and examinations under anaesthesia with cystoscopy and endoscopic biopsies at 3 monthly intervals during treatment and for 2 years from completion of treatment. Thereafter, the same examinations were carried out less frequently for up to 5 years from completion of treatment. Patients have been followed till their death or till July 1993. None has been lost to follow-up.

### Treatment Policy

After initial radiological assessment, endoscopy and biopsy diagnosis, chemotherapy was commenced. Different chemotherapy schedules were used, according to the trial current at the time each child was diagnosed (Table II). The timing and type of surgery depended on the exact site of the tumour and the initial response to chemotherapy. Radiotherapy was used either to treat microscopic post-surgical disease or as the sole local modality in cases where, in the opinion of the surgeon, an adequate resection could not be carried out without ablative surgery. More chemotherapy (9–12 months overall

Correspondence: A. Atra, Department of Haematology, Aberdeen Royal Infirmary, Foresterhill, Aberdeen AB9 2ZB, UK.
*Present address: Department of Paediatrics, St Thomas' Hospital, Lambeth Palace Road, London SE1 7EH, UK.
†Present address: Department of Morbid Anatomy, Kings College Hospital, Denmark Hill, London SE5 9RS, UK.
Received 18 August 1993; and in revised form 4 July 1994.

Table I

| Patient no. | Age at diagnosis | Site | Group | Chemotherapy | Radiotherapy (cGy) | Outcome |
|---|---|---|---|---|---|---|
| 1 (1) | 4.0 | BB/P | 3 | Intensive VAC | 5,000 | Alive: free of disease |
| 2 (2) | 6.7 | Pelvic wall | 3 | Intensive VAC | 4,440 | Alive: free of disease |
| 3 (3) | 0.75 | BB/P | 3 | VAC | 4,000 External beam brachytherapy | Alive: free of disease |
| 4 | 1.3 | BB/P | 3 | IVA | 4,000 | Alive: free of disease |
| 5 | 1.0 | V/U | 3 | Intensive VAC | 3,000 External beam brachytherapy | Died of disseminated disease |
| 6 (10) | 6.2 | BB/P | 3 | IVA | None | Alive: free of disease |
| 7 | 14.9 | Uterus | 3 | IVA | None | Alive: free of disease |
| 8 (8) | 4.3 | Pelvic wall | 3 | IVA | None | Alive: free of disease |
| 9 (5) | 2.0 | BB | 3 | VAC | 4,000 External beam brachytherapy | Alive: free of disease |
| 10 | 2.4 | Bladder dome | 1 | IVA | None | Alive: free of disease |
| 11 | 2.5 | Pelvic wall | 3 | IVA | 3,668 | Died of disseminated disease |
| 12 | 0.3 | BB/P | 3 | IVA | 4,000 | Died of disseminated disease |
| 13 (11) | 3.2 | V/U | 3 | IVA | None | Alive: free of disease |
| 14 (4) | 4.9 | BB/P | 3 | IVA | 4,000 External beam brachytherapy | |
| 15 | 2.2 | Pelvic wall | 3 | VAC/EVD | 4,500 | Died of extensive regional disease |
| 16 | 3.5 | Bladder dome | 3 | IVA | 3,000 | Alive: free of disease |
| 17 | 3.4 | Vagina | 3 | EVD | Brachytherapy only | Alive: free of disease |
| 18 | 7.1 | BB/P | 4 | VAC/EVD | 5,000 | Died of disseminated disease |
| 19 | 1.9 | BB/P | 3 | VAC/EVD | 4,000 | Alive: free of disease |
| 20 | 3.9 | BB/P | 3 | VAC/EVD | 4,400 | Alive: free of disease |
| 21 | 1.3 | Pelvic wall | 4 | Palliative care only | None | Died within 4 weeks of diagnosis |
| 22 | 0.25 | BB/P | 3 | VAC | 4,000 | Alive: free of disease |
| 23 (7) | 3.1 | Vagina | 3 | VAC | 4,500 | Alive: free of disease |
| 24 | 3.8 | BB/P | 3 | VAC/EVD | 4,000 | Died of resistant local disease |
| 25 (6) | 2.0 | BB/P | 3 | Intensive VAC | 3,000 | Alive: free of disease |
| 26 (9) | 1.5 | Bladder dome | 2 | Intensive VAC | None | Alive: free of disease |

BB/P, bladder base/prostate primary; V/U, vagina/uterus primary. For definitions of intensive VAC, VAC, IVA and VAC/EVD, see Table II.

duration) was used to treat residual disease following surgery and/or radiotherapy.

As far as 'local tumour control' is concerned, our approach was as follows. Three operative techniques were used to remove bladder tumours to try to avoid total cystectomy. These were (a) partial cystectomy, (b) submucosal resection for intravesical lesions and (c) resection of tumour masses adherent to the bladder or prostate without partial cystectomy. Total cystectomy with external diversion was used only as 'salvage therapy' for intravesical or bladder base/prostate recurrence after failure of local control by chemotherapy, radiotherapy and previous conservative surgery. The type and total dose of radiotherapy (Table III) depended on the site of the tumour and was by external beam, applicator (vaginal tumours) or urethral application of iridium wire within a Foley catheter (base of bladder/prostate lesions).

### Results

Not all of the 26 children were treated with primary chemotherapy, for the following reasons. One received only palliative care and five, including three with tumours of the bladder dome, had had primary tumour resections before referral to our hospital. The three children with bladder dome primaries survive with intact pelvic viscera after chemotherapy, although one, group 3 at diagnosis, has also received radiotherapy. The remaining 20 patients, with tumours of the bladder base and prostate (12), vagina and uterus (5) and pelvic wall (3), were treated, according to the planned treatment strategy, with primary chemotherapy followed by conservative surgery, radiotherapy or both (Figure 1). Four of these children did not come to surgery but did receive radiotherapy; two later relapsed and died of metastatic disease (one uterus/vagina primary, one pelvic wall) and two (one bladder/prostate, one uterus/vagina) survive disease free.

Table II Chemotherapy schedules $n = 25^a$

| | |
|---|---|
| **VAC** | |
| Vincristine | 1.5 mg m$^{-2}$ as bolus |
| Actinomycin D | 1.5 mg m$^{-2}$ as bolus |
| Cyclophosphamide | 1 g m$^{-2}$ as bolus |
| **Intensive VAC** | |
| Vincristine | 1.5 mg m$^{-2}$ as boluses, days 1 and 5 |
| Actinomycin D | 0.5 mg m$^{-2}$ as boluses, days 1, 3 and 5 |
| Cyclophosphamide | 500 mg m$^{-2}$ as boluses, days 1, 3 and 5 |
| **EVD** | |
| Vincristine | 1.5 mg m$^{-2}$ as bolus |
| Etoposide | 120 mg m$^{-2}$ over 4 h, days 1-3 |
| Doxorubicin | 25 mg m$^{-2}$ as bolus, days 1-2 |
| **IVA** | |
| Vincristine | 1.5 mg m$^{-2}$ as bolus |
| Actinomycin D | 1.5 mg m$^{-2}$ as bolus |
| Ifosfamide | 3 g m$^{-2}$, 3 h infusion with mesna each drug given on days 1-3 |

Chemotherapy consisted of sequential 3 weekly courses of (a) vincristine, actinomycin D and cyclophosphamide ('VAC' or 'intensive VAC') (nine patients) or (b) ifosfamide, vincristine and actinomycin D ('IVA') (ten patients) or (c) alternating 'VAC/EVD' (etoposide, vincristine and doxorubicin) (five patients). One patient received EVD alone. Courses were given every 3 weeks, or as soon afterwards as possible. Total duration of chemotherapy was 26-40 weeks. During radiotherapy, only weekly vincristine was given. [a]One child (see text) received only palliative treatment (no chemotherapy).

Sixteen children with tumours of the bladder base/prostate (11), uterus and vagina (3) and pelvic wall (2) had primary chemotherapy then local surgical resections. Of the 11 with bladder base/prostate primaries (Figure 2), two finally underwent total cystectomy with external diversion after radiotherapy had apparently failed to eradicate microscopic residual disease. One of these children died later after

**Table III** Summary of radiotherapy, according to tumour site

| *Radiotherapy* *Type* | *Dose (cGy)* | *Patient category* *Bladder prostate (n = 13)* | *Bladder dome (n = 3)* | *Uterus/vagina (n = 5)* | *Pelvic wall (n = 5)* | *Total (n = 26)* |
|---|---|---|---|---|---|---|
| External beam alone | 3,000-5,000 | 9 | 1 | 1 | 3 | 14 |
| Brachytherapy alone | 1,000 | – | – | 1 | – | 1 |
| External beam and brachytherapy | 3,000-4,000 1,000-2,000 | 3 | – | 1 | – | 4 |
| None | – | 1 | 2 | 2 | 2[a] | 7 |

[a]One palliative treatment only.

**Figure 1** Flow chart showing outcome in all 26 patients. The unique patient numbers are provided in circles, e.g. 16, and those with functional bladders are indicated.

developing lung and bony metastases; no tumour was identified in the resected specimen from the second patient, despite apparent preoperative microscopic urethral disease, and he remains disease free. Three children underwent partial cystectomy and radiotherapy. One has since died of disseminated disease. Of the two survivors, one has required an ileocystoplasty and Mitrofanoff stoma (Mitrofanoff, 1980), complicated by a vesicovaginal fistula, which was subsequently closed surgically, and the other has incomplete urinary control. Three patients underwent submucosal resections and radiotherapy. One of these children has died after local recurrence of tumour, despite subsequent prostatectomy/partial cystectomy and external beam radiotherapy. Both the survivors have incomplete urinary control. Three patients have had extravesical resections, one involving a hysterectomy. They have complete urinary control, but one has a colourethral fistula. Clearly, some patients within this group will require continence surgery in the future, and the last-mentioned child will require surgical treatment for the fistula. In addition, all three patients with uterine and vaginal primaries have undergone hysterectomy and radiotherapy, one with vaginectomy. She will also require reconstructive surgery. Two patients with tumours of the pelvic wall have undergone resection of residual masses after initial chemotherapy, one without post-operative radiotherapy, and are well without obvious functional sequelae.

There were no treatment-related deaths but, overall, 7 (27%) of the 26 children have died from their cancer (Figure 1). One child who presented with an advanced, disseminated tumour received palliative treatment only and died within 4 weeks of diagnosis. Of the remaining 25 children, four relapsed with disseminated tumour and died at a median of 12 months (range 3–41 months) from completion of initial treatment and 3 months (range 2–32 months) after treatment for relapse. One died of extensive regional disease 2 months after initial treatment and one of local disease 16 months after initial treatment. All 19 survivors are disease free from 34 to 103 months (median 71 months) from completion of treatment, and 17 retain their bladders. The other two patients (patients 4 and 9) relapsed locally 4 and 8 months from completion of initial treatment but survive 29 and 72 months after cystoprostatectomy and further chemotherapy.

Of the 12 children with bladder base/prostate primaries treated with primary chemotherapy, nine are alive and disease free; eight of these patients retain their bladder. The last adverse 'event' was 40 months from diagnosis, so most of the survivors are likely to be cured of their malignancy.

Details of bladder function in children cured of bladder/prostate primaries are provided in a separate publication (Yeung et al., 1994). The numbers of the children in Yeung's study are indicated in parenthesis in Table I. Of the four survivors from vaginal or uterus primary tumours, one had hysterovaginectomy, two had hysterectomies and one

**Figure 2** Flow chart for 11 patients with bladder base or prostate primaries and primary chemotherapy. Seven of eight survivors retained their bladder.

developed vaginal stenosis. There are therefore considerable late sequelae of this 'conservative' treatment approach.

Of the 26 children, 22 were referred for CT scans of the pelvis. Ninety-nine scans were performed, but surgical correlation, i.e. exploratory surgery carried out within 6 weeks of CT scanning, was available only in 54 instances (55%). The results of CT agreed with surgical findings in only 44 (81%) of these comparisons. In 5/10 instances of disagreement, CT suggested that tumour was present but this was not confirmed by surgery or biopsy and none of these patients has relapsed. In the five other cases there was no evidence of tumour on CT but tumour, thought to be present at surgery, was confirmed on histological examination.

Among the ten patients with bladder base/prostate lesions who achieved complete radiological remission, serial biopsies proved misleading in four. Apparently viable 'rhabdomyoblasts' were present in biopsies from three patients who have not relapsed, despite having no further treatment from 11 to 65 months (median 48 months) since the biopsy. The fourth child (already mentioned) underwent total cystoprostatectomy but no tumour was found in the resected specimen.

### Discussion

Poor control of systemic disease by chemotherapy was the main reason for treatment failure. A variety of chemotherapy regimens were used, depending upon the protocol in use at the time of diagnosis. Because of the small number of children in each subgroup, no comment on their relative efficacy can be made, and this discussion focuses on the impact of 'local' therapy (conservative surgery and radiotherapy) on treatment outcome. Our findings indicate that preservation of the bladder has not prejudiced the overall cure rate, though it is possible that the one patient who died of local disease might have benefited from earlier anterior exenteration.

There were eight treatment failures. Two patients, one with a bladder base/prostate tumour and one with a pelvic wall primary and extensive regional spread, did not achieve complete remission; both died with uncontrolled local disease. Six patients relapsed after apparent complete response. The four children with metastases all died, but two boys with only a local recurrence were 'salvaged' with further chemotherapy or radiotherapy and cystoprostatectomy.

Because of the small numbers of patients in this series, differences in outcome for the different primary sites of tumour cannot be compared for statistical significance, but three of five patients with pelvic wall tumours presented with either metastases or extensive local/regional disease and died, and a fourth child soon relapsed with metastases. Larger numbers would be needed to examine the possibility that children with primary tumours at this site present 'late' and have a relatively poor prognosis; those children with bladder and genital tract tumours tend to present with urinary symptoms and bleeding, possibly at an earlier stage (Maurer *et al.*, 1993). Primary tumours of the bladder dome are especially amenable to surgical excision (Hays *et al.*, 1990), and all three children in our study survive with good bladder function.

There is considerable morbidity among the other survivors with preserved bladders, probably the consequence of loss of functional bladder volume and sphincter damage (Yeung *et al.*, 1994). Surgery, radiotherapy, and chemotherapy all contribute. The type of morbidity depends on both the primary tumour site and the treatment approach. Some of our patients have incomplete urinary control. Those children with bladder base/prostate lesions are most likely to suffer from this complication owing to direct interference with sphincter mechanisms. Those with female genital tract primaries suffer reduction or loss of reproductive capacity. One has had a vaginectomy and one patient who had radiotherapy without surgery has vaginal stenosis. In males the local bladder base/prostate tumour and its treatment may cause impotence and sterility by interfering with autonomic nerves, the vasa deferentia and ejaculatory mechanisms, but clearly this form of morbidity is much less of an immediate handicap than total cystoprostatectomy.

Continence and reconstructive vaginal surgery is likely to be complicated by prior radiotherapy (Mclorie *et al.*, 1989). For example, the child in this series who had an ileocystoplasty later developed a vesicovaginal fistula in the tissue irradiated by external beam and iridium implant, though this has since been surgically closed, and the boy with a colovesical fistula had received radiotherapy by external beam. However, radiotherapy is an essential component of a conservative surgical policy.

Accurate assessment of local disease and its response to treatment is essential in order to plan the timing and extent of surgery. Our results demonstrate that both CT scanning and serial endoscopic assessment and biopsy are imperfect monitoring tools. Chemotherapy, radiotherapy and previous surgery may also impair interpretation. It can be difficult, for instance, to distinguish residual tumour from normal bladder muscle, both at endoscopy and on CT. Small residual tumour masses at the bladder base are not easily imaged with axial sections on CT. It is therefore not surprising to find that CT is incorrect in the assessment of residual disease in around 20% of cases. Multiplanar imaging with magnetic resonance (MRI) has been shown to be of value in the assessment of adult bladder cancer (Husband *et al.*, 1989). Ultrasound may be useful in the assessment of small residual masses and, as has been demonstrated in adults, transrectal probes provide good images of the prostate region (Brooman *et al.*, 1981). However, neither MRI nor ultrasound was assessed in this study.

Our study highlights a problem in the management of patients who, after completion of chemotherapy and/or radiotherapy, have rhabdomyoblast-like cells in endoscopic biopsies, without macroscopic evidence of recurrence. In four patients rhabdomyoblasts were present in biopsies from previously involved areas of the bladder and/or bladder neck, though no macroscopic tumour was visible at the time of biopsy. As no residual tumour was present in the resected total cystoprostatectomy specimen of the first of these patients, no additional treatment was given to the other three children and, after a median follow-up of 48 months from biopsy, none of these patient has had tumour recurrence.

This indicates that the presence of apparently viable rhabdomyoblasts in a small biopsy specimen is not necessarily an indicator of biologically malignant behaviour.

Our 'bladder salvage' rate is now much better than it was in the 1970s and early 1980s (Flamant et al., 1990) and (albeit with small numbers) superior to that of the IRS I and II studies (Maurer et al., 1988; Mclorie et al., 1989; Hays et al., 1990; Maurer et al., 1993). We accept that the preserved bladder may have impaired function and that reconstruction may be required, but our results encourage us to try to refine our current surgical conservative treatment approach rather than revert to a policy of early cystectomy/cystoprostatectomy. The close collaboration that develops between a team in a single institution, such as ours, when dealing with these patients is probably crucial. We suggest that organ preservation, with 'acceptable' function, with or without reconstructive surgery, may be an achievable goal if patients are referred to specialised centres where experience in and resources for the management of this uncommon condition are concentrated.

Six of the seven children in this series who died had uncontrollable metastatic disease, so it is evident that better chemotherapy is needed (Womer, 1993). Possibilities currently under investigation, are (a) that ifosphamide might be superior to cyclophosphamide (the 'IVA' regime rather than 'VAC') , (b) that multidrug resistance (Chan et al., 1990) might be reversed by cyclosporin or verapamil and that (c) use of 'continuous' chemotherapy, especially etoposide, might be more effective than 'pulsed' administration. Improvement in the control of systemic disease would almost certainly also boost the 'local control' rate and allow more children to complete treatment with intact, and possibly non-irradiated, pelvic organs.

Ayad Atra and Chris Mitchell were funded by the Leukaemia Research Fund. We thank Lisa Luxon for expert secretarial help and Mr Roger Brereton for allowing us to publish details of patients under his care.

### References

BROECKER, B.H., PLOWMAN, P.N., PRITCHARD, J. & RANSLEY, P.G. (1988). Pelvic rhabdomyosarcoma in children. *Br. J. Urol.*, **61**, 427-431.

BROOMAN, P.J.C., GRIFFITHS, G.J., ROBERTS, E.E., PEELING, W.B. & EVANS, K.T. (1981). Per-rectal ultrasound in the investigation of prostatic disease. *Clin. Radiol.*, **32**, 669-676.

CHAN, H.S.L., THORNER, P.S., HADDAD, G. & LING, V. (1990). Immunohistochemical detection of P-glycoprotein: prognostic correlation in soft tissue sarcoma of children. *J. Clin. Oncol.*, **8**, 689-704.

FLAMANT, F., GERBAULET, A., NIHOUL-FEKETE, C., VALTEAU-COUANET, C., CHASSAGNE, D. & LEMERLE, J. (1990). Long-term sequelae of conservative treatment by surgery, brachytherapy, and chemotherapy for vulval and vaginal rhabdomyosarcoma in children. *J. Clin. Oncol.*, **18**, 1847-1853.

HAYS, D.M., LAWRENCE, W., CRIST, W.M., WIENER, E., RANEY, R.B., RAGAB, A., TEFFT, M., WEBBER, B., JOHNSTON, J. & MAURER, H.M. for the Intergroup Rhabdomyosarcoma Study (1990). Partial cystectomy in the management of rhabdomyosarcoma of the bladder: a report from the Intergroup Rhabdomyosarcoma Study. *J. Pediatr. Surg.*, **25**, 719-723.

HUSBAND, J., OLLIFF, J.F., WILLIAMS, M.P., HERON, C.W. & CHERRYMAN, G.R. (1989). Bladder cancer: staging with CT and MR imaging. *Radiology*, **173**, 435-440.

McLORIE, G.A., ABARA, O.E., CHURCHILL, B.M., GREENBERG, M. & MANCER, K. (1989). Rhabdomyosarcoma of the prostate in childhood: current challenges. *J. Pediatr. Surg.*, **24**, 977-981.

MAURER, H.M., BETTANGADY, M., GEHAN, E.A., CRIST, W., HAMMOND, D., HAYS, D.M., HEYN, R., LAWRENCE, W., NEWTON, W., ORTEGA, J., RAGAB, A.H., RANEY, R.B., RUYMANN, F.B., SOULE, E., TEFFT, M., WEBBER, B., WHARAM, M. & VIETTI, T.J. (1988). The Intergroup Rhabdomyosarcoma Study I. *Cancer*, **61**, 209-220.

MAURER, H.M., GEHAN, E.A., BETTANGADY, M., CRIST, W., DICKMAN, P.S., DONALDSON, S., FRYER, C., HAMMOND, D., HAYS, D.M., HERRMANN, J., HEYN, R., MORRIS JONES, P., LAWRENCE, W., NEWTON, W., ORTEGA, J., RAGAB, A.H., RANEY, R.B., RUYMANN, F.B., SOULE, E., TEFFT, M., WEBBER, B., WIENER, E., WHARAM, M. & VIETTI, T. (1993). The Intergroup Rhabdomyosarcoma Study II. *Cancer*, **71**, 1904-1922.

MITROFANOFF, P. (1980). Cystostomie continente trans-appendiculaire dans le traitement des vessies neurologiques. *Chir. Pédiatr.*, **21**, 297-305.

RANEY, R.B., GEHAN, E.A., HAYS, D.M., TEFFT, M., NEWTON, W.A., HAEBERLEN, V. & MAURER, H.M. (1990). Primary chemotherapy with or without radiation therapy and/or surgery for children with localized sarcoma of the bladder, prostate, vagina, uterus and cervix. *Cancer*, **66**, 2072-2081.

WOMER, R.B. (1993). The Intergoup Rhabdomyosarcoma Study I. *Cancer*, **71**, 1719-1721.

YEUNG, C.K., WARD, H.C., ATRA, A., RANSLEY, P.G., PLOWMAN, P.N., DUFFY, P.G. & PRITCHARD, J. (1994). Bladder and kidney function after cure of pelvic rhabdomyosarcoma in childhood (submitted).


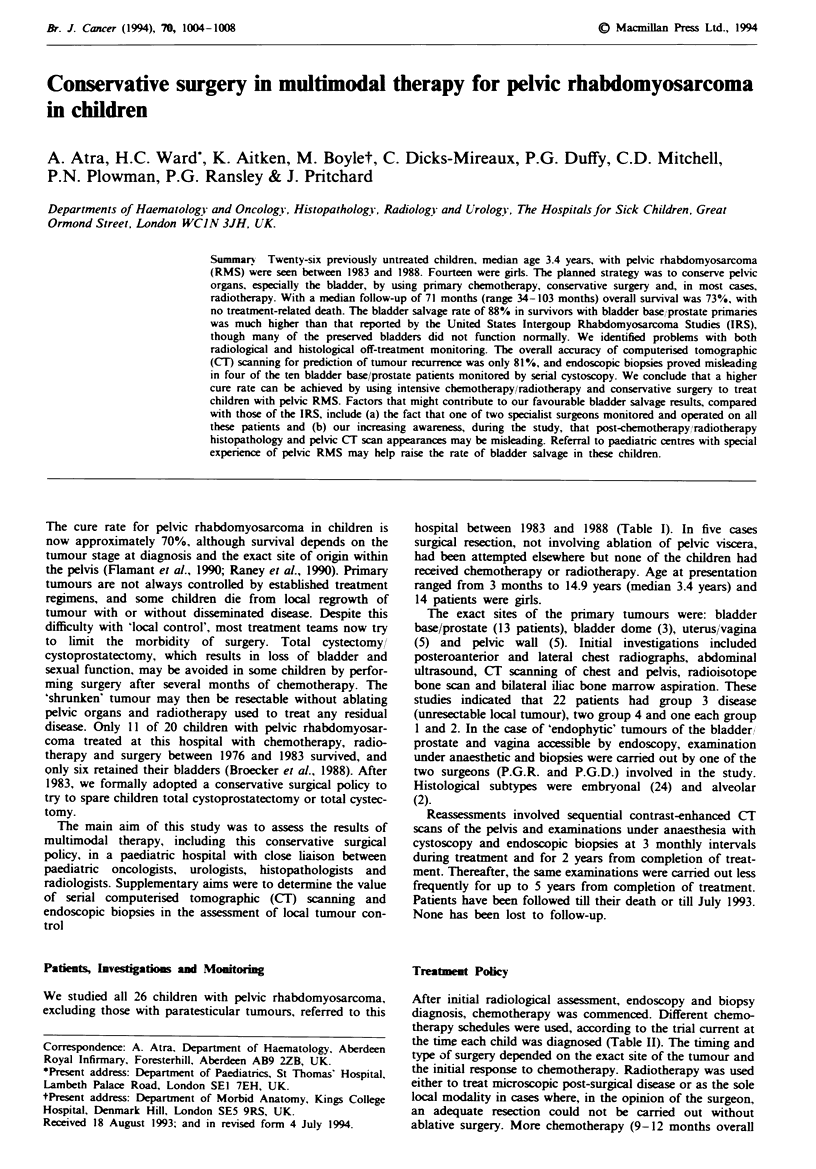

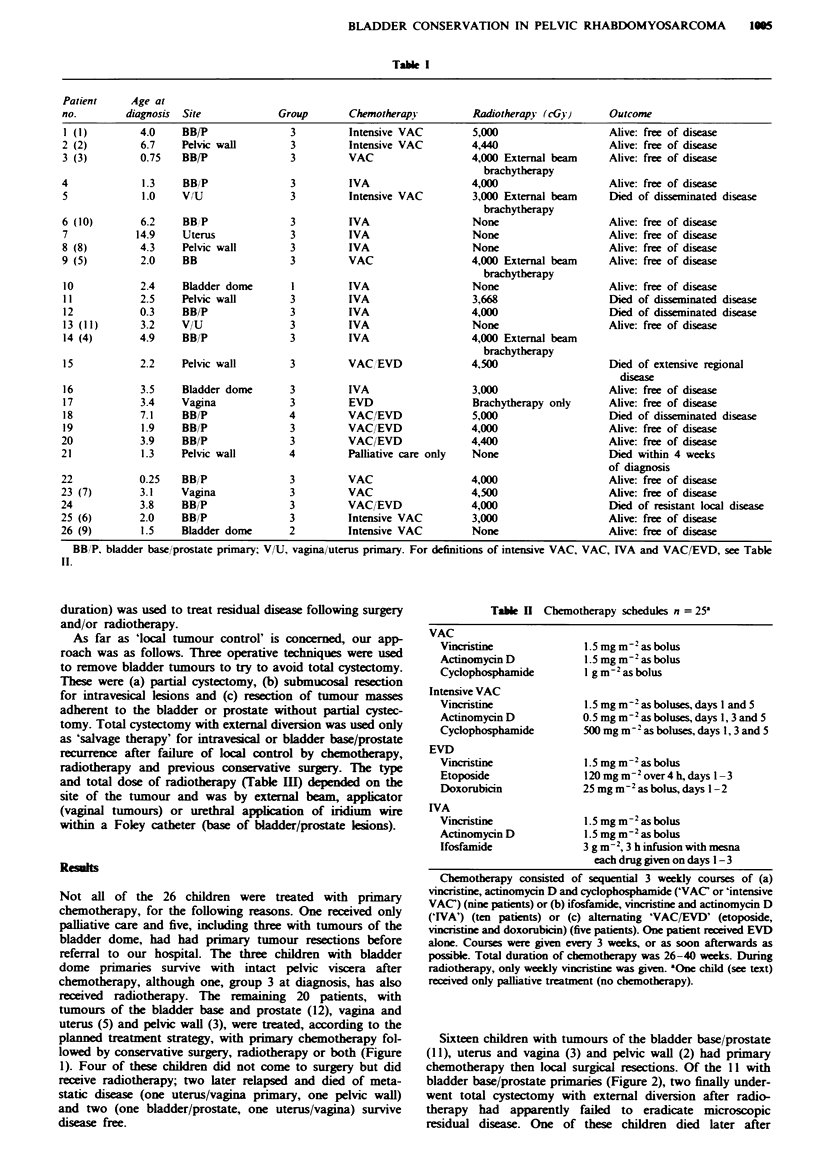

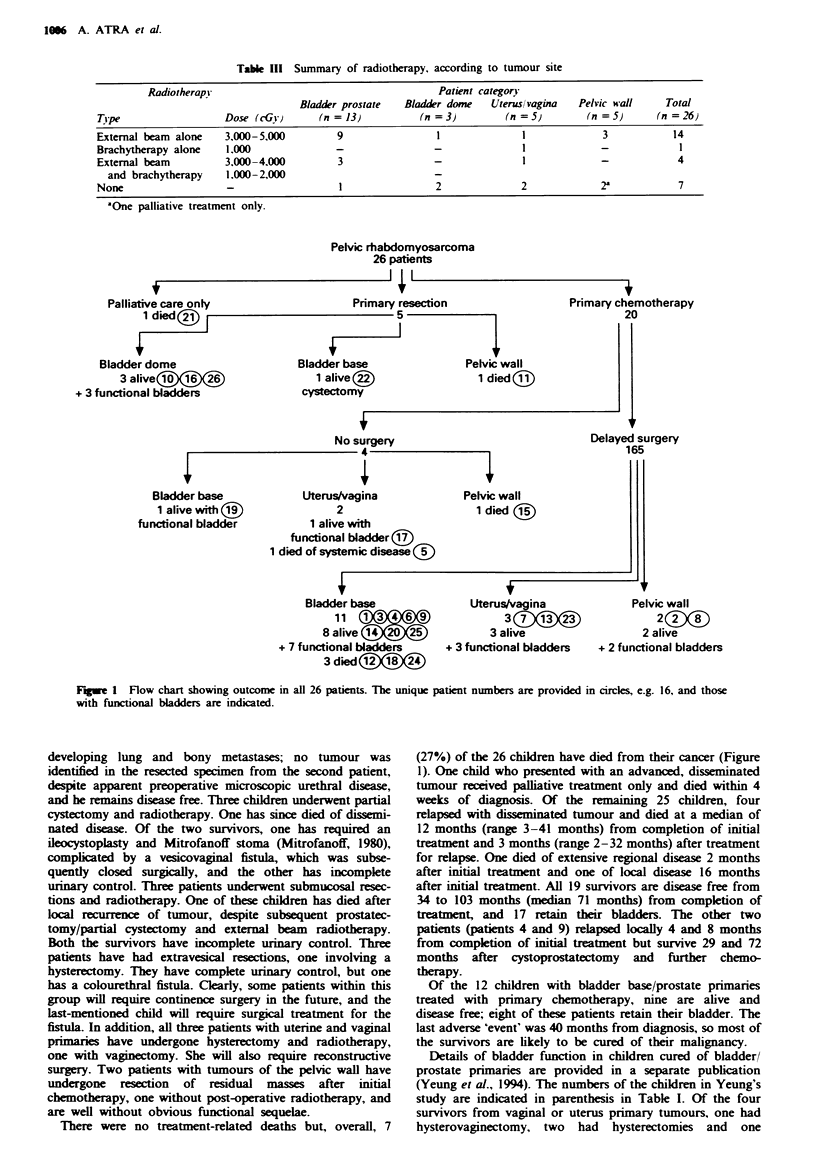

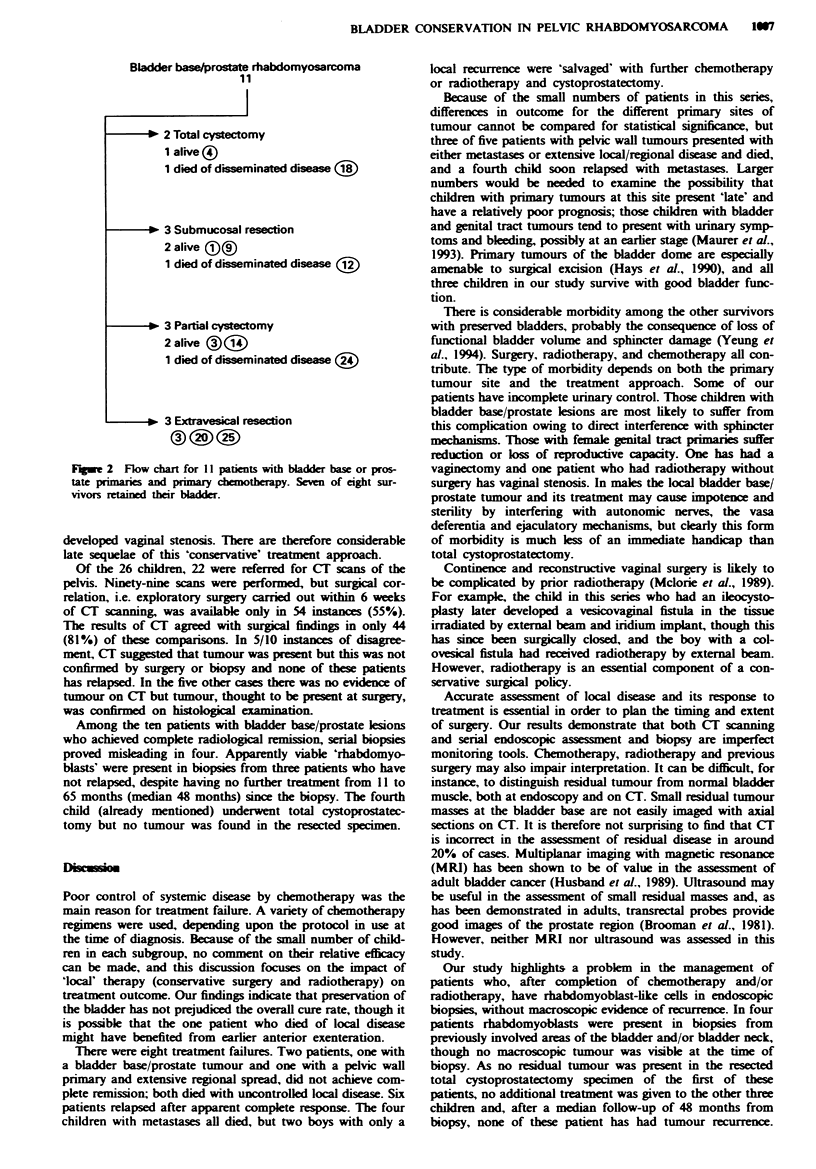

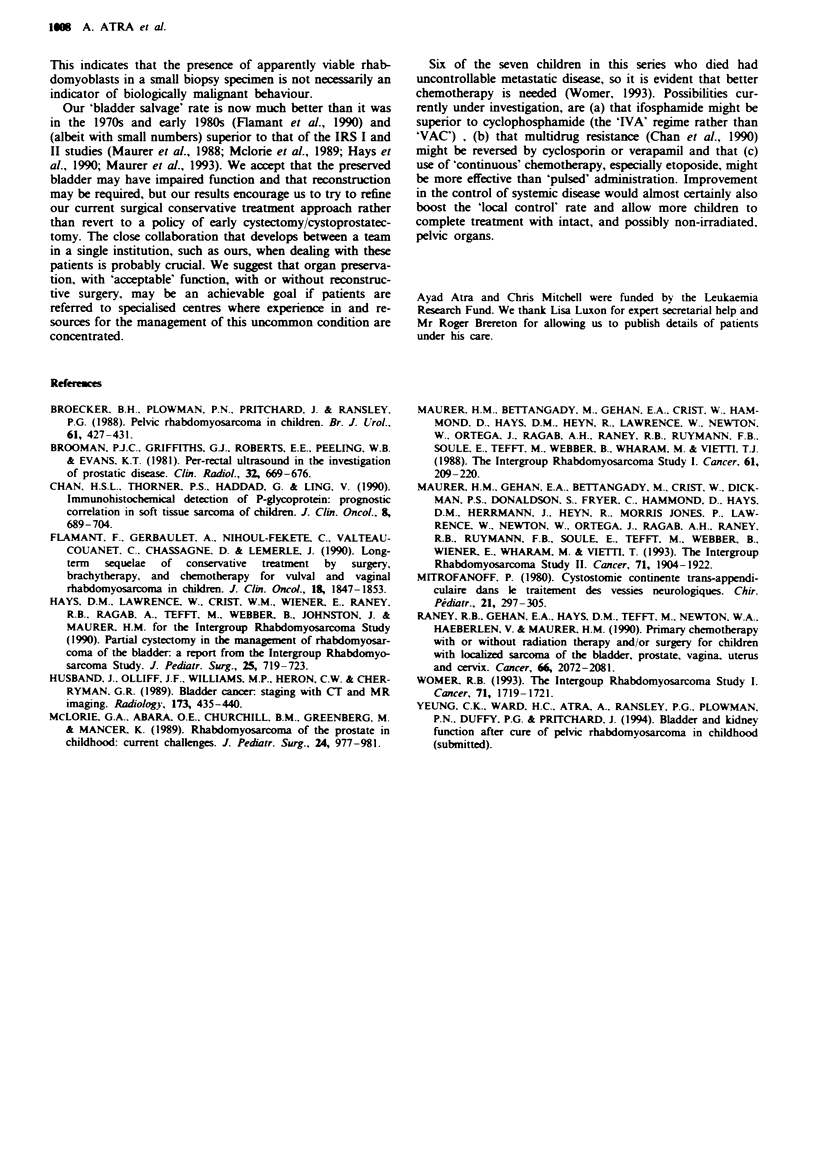


## References

[OCR_00869] Broecker B. H., Plowman N., Pritchard J., Ransley P. G. (1988). Pelvic rhabdomyosarcoma in children.. Br J Urol.

[OCR_00874] Brooman P. J., Griffiths G. J., Roberts E., Peeling W. B., Evans K. (1981). Per rectal ultrasound in the investigation of prostatic disease.. Clin Radiol.

[OCR_00879] Chan H. S., Thorner P. S., Haddad G., Ling V. (1990). Immunohistochemical detection of P-glycoprotein: prognostic correlation in soft tissue sarcoma of childhood.. J Clin Oncol.

[OCR_00887] Flamant F., Gerbaulet A., Nihoul-Fekete C., Valteau-Couanet D., Chassagne D., Lemerle J. (1990). Long-term sequelae of conservative treatment by surgery, brachytherapy, and chemotherapy for vulval and vaginal rhabdomyosarcoma in children.. J Clin Oncol.

[OCR_00892] Hays D. M., Lawrence W., Crist W. M., Wiener E., Raney R. B., Ragab A., Tefft M., Webber B., Johnston J., Maurer H. M. (1990). Partial cystectomy in the management of rhabdomyosarcoma of the bladder: a report from the Intergroup Rhabdomyosarcoma Study.. J Pediatr Surg.

[OCR_00914] Maurer H. M., Beltangady M., Gehan E. A., Crist W., Hammond D., Hays D. M., Heyn R., Lawrence W., Newton W., Ortega J. (1988). The Intergroup Rhabdomyosarcoma Study-I. A final report.. Cancer.

[OCR_00905] McLorie G. A., Abara O. E., Churchill B. M., Greenberg M., Mancer K. (1989). Rhabdomyosarcoma of the prostate in childhood: current challenges.. J Pediatr Surg.

[OCR_00927] Mitrofanoff P. (1980). Cystostomie continente trans-appendiculaire dans le traitement des vessies neurologiques.. Chir Pediatr.

[OCR_00934] Raney R. B., Gehan E. A., Hays D. M., Tefft M., Newton W. A., Haeberlen V., Maurer H. M. (1990). Primary chemotherapy with or without radiation therapy and/or surgery for children with localized sarcoma of the bladder, prostate, vagina, uterus, and cervix. A comparison of the results in Intergroup Rhabdomyosarcoma Studies I and II.. Cancer.

[OCR_00939] Womer R. B. (1993). The Intergroup Rhabdomyosarcoma Studies come of age.. Cancer.

